# Diphtheria of the Conjunctiva

**Published:** 1904-08-13

**Authors:** 


### DIPHTHERIA OF THE CONJUNCTIVA.

Mr. Sydney Stephenson,[1] in reporting a case of diphtheria of the conjunctiva, states that the disease is far from rare in London. In 3,412 cases of eye disease seen at children's hospitals, 43 instances were noted. In only three of these, it is true, were the local conditions such as are usually described in text-books under the term "diphtherial conjunctivitis." But, both in these and in others of "mild" or "medium" type, the Klebs-Löffler bacillus was shown to be present, and the author argues that the extreme forms at either end of the series are linked together by intermediate grades. Regarding other clinical evidences of the disease, it is noted that in

40 per cent. of the cases the children showed such signs of constitutional disturbance as pallor, anorexia, languor, or wasting, with, for the most part, a subnormal rather than an elevated temperature. In some albumen was present in the urine, in others the knee-jerks were absent. In the more severe cases the pre-auricular and angular glands were enlarged, but in the mildest cases no such enlargement existed. The fauces and nostrils were involved only in a few instances. In 32 out of the 43 patients only one eye was affected. In all degrees of the disease antitoxin has a definite influence.

[1] The Ophthalmoscope, August 1904.

